# Effects of a Diabetic Microenvironment on Neurodegeneration: Special Focus on Neurological Cells

**DOI:** 10.3390/brainsci14030284

**Published:** 2024-03-15

**Authors:** Vishal Chavda, Dhananjay Yadav, Snehal Patel, Minseok Song

**Affiliations:** 1 Pharmacology Research Lab, Department of Pharmacology, Nirma University, Ahmedabad 382481, India; chavdavishal2@gmail.com; 2 Department of Life Science, Yeungnam University, Gyeongsan 38541, Republic of Korea; dhanyadav16481@gmail.com

**Keywords:** astrocytes, diabetes, metabolic complications, glial dysfunction, neurodegeneration

## Abstract

Diabetes is a chronic metabolic condition associated with high levels of blood glucose which leads to serious damage to the heart, kidney, eyes, and nerves. Elevated blood glucose levels damage brain function and cognitive abilities. They also lead to various neurological and neuropsychiatric disorders, including chronic neurodegeneration and cognitive decline. High neuronal glucose levels can cause drastic neuronal damage due to glucose neurotoxicity. Astrocytes, a type of glial cell, play a vital role in maintaining brain glucose levels through neuron–astrocyte coupling. Hyperglycemia leads to progressive decline in neuronal networks and cognitive impairment, contributing to neuronal dysfunction and fostering a neurodegenerative environment. In this review, we summarize the various connections, functions, and impairments of glial cells due to metabolic dysfunction in the diabetic brain. We also summarize the effects of hyperglycemia on various neuronal functions in the diabetic brain.

## 1. Introduction

The central nervous system (CNS) controls most bodily functions and maintains normal brain functions such as physical support, formation of the blood–brain barrier (BBB), regulation of cerebral blood flow and synaptic transmission, neurotransmitter uptake, and energy storage and transport [1]. Neurons and glial cells are fundamental components of the nervous system. Neurons generate and transmit electrical and chemical signals, and glial cells chiefly modulate neuronal function and signaling [2]. Similar to neurons, glial cells (which are non-neuronal cells) consist of various types. They are equipped with a diverse range of functions, including modulation of synapse formation, function, and elimination; synthesis of myelin; regulation of blood flow and metabolic molecules; and maintenance of ionic and water homeostasis [3]. They also deliver nutrients to nourish neurons, provide anatomical support for CNS development, and contribute to the establishment of synaptic connections and pruning [4,5,6,7]. Astrocytes, oligodendrocytes, ependymal cells, radial glia, and microglia are different types of glial cells found in the CNS, while Schwann cells, satellite cells, and enteric glia are specific to the peripheral nervous system (PNS) [8,9,10,11,12]. These distinct cell types vary in morphology, anatomical location, developmental origin, physiological function, and molecular composition [13]. 

The CNS has two major types of glial cells: microglia and macroglia. These have an array of different functions, being crucial for maintaining homeostatic neuronal circuits [14]. Macroglia comprise astrocytes, oligodendrocytes, tanycytes, and ependymal cells [15,16]. Hypothalamic microglia, astrocytes, and tanycytes control the signals of metabolic neuronal circuits to regulate both physiological and pathophysiological mechanisms involved in appetite and metabolism. Glial cells secrete and deliver gliotransmitters, hormones, growth factors, cytokines, and nutrients and serve as a forerunner for neuroprogenitor cells [17]. They actively participate in neurophysiological processes including neurogenesis, synaptogenesis, synaptic plasticity, synaptic transmission, and neuronal survival [18]. Glial cells are the earliest defenders of the CNS, as they substantially promote the preservation of BBB integrity. Their presence throughout the CNS elicits diverse neuronal responses depending on their discrete glial populations in the local network of neurons [19,20]. 

It has been reported that hyperglycemia, which is a state of high blood glucose in the body, elevates the risk of causing dyslipidemia, hypertension, and complications of kidney dysfunction, retinopathy, and neuropathy [21,22,23]. The principal cause of diabetes is insulin resistance, which occurs when bodily tissues (insulin-sensitive tissues such as the liver, adipose tissue, and muscles) do not respond appropriately to insulin, causing blood glucose to rise [24,25]. Oxidative stress and inflammation are the two most important contributors to the development and progression of type 2 diabetes (T2D) [26,27,28]. Epidemiological studies have confirmed that patients with T2D perform less well than controls in terms of the cognitive domains of information-processing speed, memory, attention, and execution function [29,30,31]. According to Moran et al., patients with T2D have acquired brain atrophy and lower total gray, regional white, and hippocampal volumes. They discovered that brain volume decrease was associated with poor cognitive function in these type 2 diabetic patients [32]. Animal models of insulin deficiency and chronic hyperglycemia have already been proposed, showing a variety of metabolic abnormalities, spatial learning, synaptic plasticity impairments, synaptic degeneration, increased astrocyte responsiveness, proliferation, and oxidative stress [33,34,35,36,37]. Hence, in this work, we highlight various glial cells, particularly astrocytes, their roles, and the damage caused by metabolic dysfunction in the diabetic brain. Additionally, we investigate the effects of hyperglycemia on neural activity in the diabetic brain.

## 2. Astrocytes

Brain tissues require a substantial amount of energy; thus, a well-organized machinery and complex mechanisms are employed to operate and ensure the adequate spatiotemporal delivery of energy substrates accompanying neuronal activity [38]. Astrocytes possess fine processes with a nonoverlapping, highly ramified “tiled” structure, constituting the astrocyte territory in the healthy brain [39]. These are star-shaped, plentiful, and versatile cells that facilitate synaptogenesis, synaptic connectivity, signaling, and anatomical neural support. They contribute to neuronal protection and survival, and they also regulate and maintain nutrient entry, metabolism, energy homeostasis, electrolytes, cytokines, and neurotransmitter production within the brain [40,41]. As part of the neurovascular unit, they form vascular processes known as end feet. These end feet intimately contact the neurovasculature, mainly interacting with intraparenchymal blood vessels. At synapses, astrocytes ensheath neurons through their fine perisynaptic processes. These perisynaptic processes express a wide range of receptors for neurotransmitters, growth factors, cytokines, and various ion channels and transporters [38]. The main role of neurons is to release neurotransmitters, which in turn correspondingly activate astrocyte receptors. This activation triggers a Ca^2+^ response, promoting the secretion of vasoactive substances in the astrocytic end feet. These structures come into contact with cerebral blood vessels, thereby promoting cerebral blood flow and oxygen supply [42,43,44]. The function of astrocytes is not limited to increasing blood flow and local oxygenation; they are also leading energy producers, utilizers, and distributors in the brain. Astrocytes expand their duties by taking up blood glucose via glucose transporter 1 (GLUT 1 transporter) [45], converting it into lactate, and then releasing it to neurons for local energy use [46,47]. In the CNS, they function as the sole fatty acid oxidizers and ketone body producers that act as specific signals for nearby neurons and govern neuronal metabolic sensing. Therefore, astrocytes serve as sensory mediators, linking neurons and blood vessels to interconnect metabolic and synaptic transmission between the CNS and PNS [20]. To fulfill glucose- and lipid-sensing purposes, astrocytes express receptors and respond to appetite regulator hormones such as insulin, leptin, and ghrelin. As a result, hormones and metabolic factors related to the exposure time reshape astrocytic morphology in terms of the length and number of primary projections. These changes successively modulate glial coverage, synaptic outputs, the release of cytokines, and the transportation of glutamate and glucose, leading to various physiological effects. 

Astrocytes are dynamic, active partners of neurons that process, integrate, and transmit synaptic information and plasticity [48,49]. Glutamatergic synapses are essential for transmitting signals between neurons and are crucial for learning and memory. Glutamate, a primary excitatory neurotransmitter in the brain, can induce excitotoxicity if its receptors are overstimulated, posing a highly toxic event for neurons. Astrocytes play an important role in synaptic transmission, not only through the rapid release of neurotransmitters into the synaptic cleft but also by ensuring the termination of synaptic transmission and maintaining neuronal excitability. They predominantly take up glutamate via astrocyte-specific, Na^+^-dependent, high-affinity glutamate transporters. These transporters include the excitatory amino acid transporter 1, commonly known as glutamate aspartate transporter 1, and excitatory amino acid transporter 2, often referred to as glutamate transporter 1. Astrocytes maintain neuronal excitability by preserving the neurotransmitter pool of glutamate through a cascade of glutamate–glutamine cycles [50]. The glutamate–glutamine cycle occurs between neurons and astrocytes. Primarily, neurons release glutamate, which is taken up by astrocytic glutamate transporters. Subsequently, the astrocyte-specific enzyme glutamine synthetase converts glutamate to glutamine. Astrocytes then transfer glutamine to neurons, where it is converted back to glutamate by glutaminase. Additionally, the tricarboxylic acid cycle contributes to the glutamate–glutamine cycle in astrocytes and neurons [51]. Astrocytes are the sole glutamate replenishers in the brain, owing to their expression of the unique enzyme pyruvate carboxylase. This enzyme facilitates the synthesis of glutamate from glucose through an anaplerotic reaction [38]. 

Prolonged exposure to intense, unfavorable stimuli, such as a high-fat diet (HFD) intake or persistent obesity, can substantially alter astrocyte structure and modulate the production and transport of nutrients, cytokines, and neurotransmitters. Astrocytes become reactive, and their atypical proliferation or astrogliosis induces cytoskeletal hypertrophy. This is characterized by the expression of extracellular matrix molecules such as chondroitin sulfate proteoglycans, fibronectin, and laminins, along with an enhanced expression of the glial fibrillary acidic protein [52]. However, astrocyte-mediated cytokine secretion has a dual nature, as stimulus type, potency, and duration can have positive or negative effects [20,53]. Thus, astrogliosis is a reactive event usually observed in astrocytes following CNS insult [54]. The following section describes hyperglycemia, which may cause cognitive impairment as a result of malfunctioning astrocytes.

## 3. Hyperglycemia and Cognitive Decline Due to Astrocyte Dysfunction

Hyperglycemia adversely affects the cerebral vasculature and promotes cognitive deterioration and dementia [55]. Insulin regulates energy metabolism, which in turn influences glucose homeostasis, redox balance, osmolarity, synaptic plasticity, learning, and memory. Glucose catabolism via glycolysis is a fundamental pathway for energy production and neurotransmission in the mature brain. This pathway is facilitated by the glutamine-glutamate/GABA cycle between astrocytes and neurons. Moreover, the rate of glutamatergic and GABAergic neurotransmission depends on the extracellular glucose concentration. Defective astrocyte glucose metabolism attenuates memory consolidation [56]. Furthermore, astrocytes possess a considerable amount of various reactive oxygen species (ROS)-detoxifying enzymes and antioxidant molecules, including glutathione, glutathione peroxidase, glutathione S-transferase, thioredoxin reductase, heme-oxygenase 1, and catalase [38,57,58]. Fortunately, astrocytes are appreciably more resistant to cellular damage induced by pro-oxidant molecules such as hydrogen peroxide, nitric oxide, peroxynitrite, and 6-hydroxy dopamine owing to their high antioxidant potential [59]. For example, astrocytes counteract nitric oxide by promoting the glycolytic pathway to increase glucose oxidation and limit extracellular adenosine triphosphate (ATP) levels to prevent the intrinsic pathway of apoptosis. As astrocytes benefit from this mechanism, they utilize their antioxidant competency to safeguard nearby neurons from toxic pro-oxidant compounds and oxidative stress [60,61]. Hence, faithfully performing homeostatic and neuroprotective functions under normal physiological conditions may endow astrocytes with a double-edged sword under pathological conditions. Astrocytes are also capable of influencing synaptic transmission through the Ca^2+^-dependent release of gliotransmitters, including d-serine, glutamate, ATP, and adenosine [38]. In the normal state, astroglial cells control glutamate and water movement and distribution, protect against oxidative stress, store glycogen as an energy source, facilitate tissue repair, buffer extracellular potassium ion levels, and modulate synaptic activity. This modulation occurs through the release of gliotransmitters, neurotrophic factors, and the regulation of extracellular concentrations of neurotransmitters released from adjoining synapses [5,48,60]. Astroglial cells also play a vital role in metabolic support and the neutralization of ROS generated in vicinal neuronal mitochondria. Hence, under pathological conditions, astrocytes become disabled, subsequently causing brain edema [62,63] due to the lack of control over water movement and the production of toxic biomolecules such as hydroxyl radicals, ROS, nitric oxide, and peroxynitrite [64]. Moreover, stressed astrocytes can overturn neurotransmitter transporters by releasing glutamate, and cytotoxic levels of Ca^2+^ and intracellular Ca^2+^ promote neural demise. Sick astrocytes fail to buffer extracellular K^+^ accumulation that successively overexcites neuronal cells [65,66]. The appropriate functioning of astroglial cells is crucial for brain homeostasis, synaptogenesis, and synaptic pruning [67]. Astrocytic dysfunction substantially contributes to neurological deficits and degeneration processes [68]. Evidence suggests that amplifying or reestablishing astrocytic functions may constitute promising therapeutic avenues for neurodegenerative disorders, as it is currently a burgeoning field.

**Figure 1:**
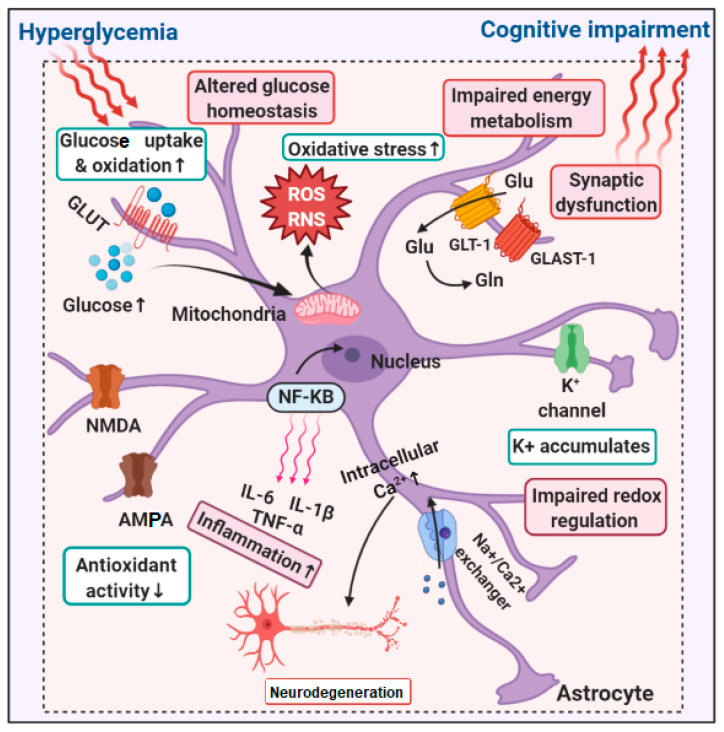
Alterations in astroglia associated with hyperglycemia and cognitive impairment. GLUT, glucose transporter; NMDA, N-methyl D-aspartate; AMPA, α-amino-3-hydroxy-5-methyl-4-isoxazolepropionic acid; Glu, glutamate; Gln, glutamine; GLAST-1, L-glutamate/L-aspartate transporter.

The hyperglycemic milieu induces various unfavorable phenomena that adversely affect astrocyte structure and function. High glucose levels enhance mitochondrial oxidative stress due to increased glucose oxidation and increased production of ROS and reactive nitrogen species. This leads to altered glucose homeostasis and impaired energy metabolism in astrocytes. Oxidative stress also modulates the activation of NF-κB, the master transcription regulator of proinflammatory cytokines, resulting in inflammation. Hyperglycemia depletes the antioxidant enzymes in astrocytes, thereby ameliorating their antioxidant properties and influencing the conversion of glutamine to glutamate in astrocytes. In turn, astrocytic Ca^2+^ and K^+^ concentrations are altered, with toxic levels triggering neurodegeneration and synaptic dysfunction, thereby inducing cognitive impairment (Figure 1).

Recent neuroenergetic studies have highlighted the implications of the cooperative interplay between astrocytes and neurons [69,70,71,72]. Major processes responsible for the high energy expenditure in the brain include the maintenance and restoration of electrolyte gradients dissipated by action potentials, postsynaptic signaling events, and the uptake and recycling of neurotransmitters [38,73]. Astrocyte–neuron coupling plays vital roles in the energy metabolism of the brain. Astrocytes are essential components of the BBB, continuously transporting glucose, which shows their direct role in the regulation of metabolism. The metabolic coupling between astrocytes and neurons is crucial for maintaining physiological homeostasis, as it regulates antioxidant defenses, inflammatory responses, innate immunity, and energy metabolism [74,75,76]. Hyperglycemia dramatically modulates the function of astrocytes in glucose metabolism, inhibiting their proliferation and migration and inducing cell cycle arrest by reducing levels of cyclin D1 and D3 in primary astrocytes. Due to the prolonged high blood glucose milieu, gap junction communication becomes irreversibly impaired. This impairment elevates the activation of the adenosine monophosphate (AMP)-activated protein kinase signaling pathway, leading to an increase in glycolysis and ATP, as well as elevated levels of ROS and inflammatory cytokine expression. Consequently, there is an induction of increased cell apoptosis in primary astrocytes, impacting both energy metabolism and the functional phenotype of astrocytes. These findings strongly suggest the involvement of astrocytes in diabetic cerebral neuropathy [54]. Astrocytes can adapt to energy metabolic changes to withstand cellular challenges and have a higher metabolic plasticity than neurons [38].

## 4. Microglia

The most prominent mononuclear macrophages of the CNS are microglial cells. They continuously patrol the brain to eliminate cellular debris and pathogens, serving as the first line of defense against various infections to preserve a healthy brain. Microglial cells maintain CNS health by regulating key aspects of synapse development, function, and plasticity and refining and sculpting synaptic circuits by abolishing excess and unwanted synapses [77]. These self-regenerative cells [78] are the most flexible glial cells; they behave like chameleons, as they can modulate their morphological state and biological functions according to the surrounding environment. Under normal physiological conditions, they take on the shape of “resting or ramified microglia” and harmonize synapse input and output. They produce essential cytokines to mediate brain immunological responses and facilitate synaptic pruning through the complement system [5,79]. Under unhealthy conditions that arise due to overfeeding, overnutrition, or HFD consumption, microglial cells become reactive, adopting a macrophage-like (ameboid) structure and releasing diverse factors in response to various stimuli [5]. Morphological shifts in microglia occur due to dynamic activation through crosstalk among neurons, astrocytes, and microglial cells, essentially benefiting adaptive neuroplasticity [80]. In particular, an HFD, rich in saturated fatty acids, stimulates hypothalamus-bearing microglia to release inflammatory cytokines and toxic anions, mainly ROS and reactive nitrogen species [81]. Microglia-responsive neurons and hypothalamic pro-opiomelanocortin neurons are particularly vulnerable to metabolic changes or neuropeptides associated with metabolism. This vulnerability arises due to their heightened sensitivity to the chronic secretion of inflammatory and oxidative stress factors, resulting in toxicity in the autonomic nervous system. Consequently, the interplay between microglial cells and metabolic neurons plays a crucial role in modulating chemical radical and cytokine production, contributing to the establishment of an inflammatory milieu. These neurotoxic events can promote the development of insulin resistance, leptin resistance, glucose intolerance, hyperglycemia, obesity, and other metabolic disorders [20]. Furthermore, activated microglia can cause transganglionic degeneration due to dysregulated immune responses and degenerative events [82]. However, it is not merely a disturbance in the physiological functioning of microglial cells, which mainly involves synaptic regulation, that may lead to neurological disorders. More accurately, these can be designated as “microglia-neuron coupling disorders” instead of defective neuronal functioning. Various cognitive deficits are likely related to dysregulated microglial synaptic pruning, suggesting a potential therapeutic target in these disorders [77,83,84]. Thus, microglial cells are well known for their immunological and homeostatic roles in the CNS; however, understanding their complete role is urgently needed [85]. 

## 5. Myelinating Glia: Oligodendrocytes and Schwann Cells

This intricate nervous system contains myelinated neurons closely associated with oligodendrocytes in the CNS and Schwann cells in the PNS. Certainly, a single oligodendrocyte may contact and myelinate several axons in the CNS [86], while a single Schwann cell myelinates only one axon in the PNS, forming a “long beads on a string” organization, where one axon is ensheathed by many Schwann cells. Both glial cell types arise distinctly; Schwann cells originate from neural crest derivatives, whereas cerebral and spinal cord oligodendrocytes arise from the subventricular zone and ventral neural tube precursors, respectively [87,88]. Close proximal crosslinking of myelinating glia and axons can also disrupt the subject, leading to demyelination. This demyelination may occur in response to acute injuries or diseases resulting from genetic factors, autoimmunity, metabolic impairment, and mechanical or ischemic insults [89,90]. Only limited knowledge has been gained on myelinating glia, which calls for immediate and effective insights into the underlying pathophysiological mechanisms of these vital glial cells. 

Diabetes-induced alterations in Schwann cells are linked to neuronal damage, leading to diabetic peripheral neuropathy [91,92]. These alterations compromise the synthesis and release of neuronal support factors and result in the build-up of neurotoxic and pro-inflammatory factors, including tumor necrosis factor (TNF-α), interleukin (IL)-1α, IL-1β, monocyte chemoattractant protein-1 (MCP-1), and CXCL2, which in turn contribute to endothelial dysfunction, axonal degeneration, and neuronal damage [93,94,95]. Segmental demyelination is a major hallmark of the symmetric polyneuropathy of diabetic individuals, most likely related to Schwann cell disruption [96,97]. One study investigated the remyelinating behavior of both cell types, namely Schwann cells and oligodendrocytes, after ethidium bromide (EB) injection into the brainstem of streptozotocin-treated diabetic rats. This animal model had previously been used to study myelinations in the peripheral nervous system occurring in diabetic patients [98]. The study found that diabetic rats had delayed macrophage activity and poorer remyelination than non-diabetic rats. Collectively, the study concluded that diabetes may hinder both oligodendrocyte and Schwann cell remyelination compared to non-diabetic rats. 

Wang and colleagues recently investigated whether hyperglycemia-induced neurobehavioral deficits were associated with the dysfunction of oligodendrocyte precursor cells (OPCs) in mice [99]. OPCs react to myelin injury by developing into myelinating oligodendrocytes; however, in diabetic mice, this response appears to be hindered, exhibiting poor oligodendrogenesis, exacerbating symptoms, and exhibiting neuronal damage seen in other events like stroke. The authors revealed that the quantity of OPCs and the amount of basic myelin protein expression were reduced in diabetic mice, and migratory and survival abilities were severely repressed in a hyperglycemic environment in vitro. Hence, the study may suggest that diabetes-induced neurological impairments were connected with a decrease in the quantity and functioning of OPCs [99]

## 6. Tanycytes

Tanycytes are specialized ependymal cells that occupy the third ventricle of the hypothalamus. This subtype of glial cells consists of ependymal cells, which are composed of simple cuboidal secretory cells. These cells line the ventricles of the brain and the central canal of the spinal cord, playing a regulatory role in the production of cerebrospinal fluid. They have long processes and end feet that connect with proximal neurons and blood capillaries in the brain. Tanycytes can be categorized into α and β types, which are distinct both in their compartmentalization and biological activities [100]. Tanycytes securely attach to endothelial cells via tight junctions, forming a BBB that finely tunes and maintains the diffusion of blood-borne molecules into the cerebrospinal fluid. Their special localization, with unique accessibility to substances in the brain, allows us to identify them as key regulators of the CNS [101,102]. Tanycytes are also capable of regulating nutrient and hormone responses that reach metabolic neuronal circuits in the hypothalamus, ultimately influencing metabolism, appetite, and body weight. Hence, the communication between cerebrospinal fluid and metabolic neuronal circuits, which is dependent on nutrition and hormones, is enabled by the long processes of these glial cells in the arcuate and ventromedial nuclei of the hypothalamus. Tanycytes adapt their function based on metabolic conditions to control glucose homeostasis by expressing GLUT1, GLUT2 transporters, and glucokinase [103,104]. Additionally, they regulate BBB permeability by secreting vascular endothelial growth factor A to promote the transmission of circulating metabolites in metabolic neurons. Specific hormones and metabolic signals can trigger several downstream signaling pathways in the hypothalamus. However, different metabolic-associated conditions, such as hypoglycemia, chronic HFD-induced obesity, insulin intolerance, and hyperglycemia, can induce contrasting signaling events and outcomes. For instance, leptin activates the ERK signaling pathway, and insulin and insulin-like growth factor 1 (IGF-1) initiate a conventional intracellular signaling pathway that forms an interconnected link between metabolic modulation and cognition [20,105].

According to recent research, abnormal tanycytic function may be the cause of metabolic disorders, including T2D and obesity, by obstructing peripheral signals’ ability to reach the hypothalamic circuits that control energy homeostasis and metabolism [106,107]. Interestingly, these defects are also linked with an increased risk of developing Alzheimer’s disease (AD), suggesting that tanycytes could represents the missing link connecting T2D, obesity, and AD. [108]. Tanycytic pathology is also associated with adequate neuroendocrine functioning. It is known that the median eminence of hypothalamus tanycytic end feet interacts with the terminals of neuroendocrine neurons to modulate the secretion of neurohormones via hypothalamic–pituitary axes [109,110].

## 7. Hyperglycemic Brain

Hyperglycemia can induce glucose neurotoxicity, a phenomenon that increases neuronal glucose levels and triggers neuronal damage or neuropathy [111]. In the human body, the brain is undoubtedly the most expensive organ in terms of its strict glucose dependency on energy expenditure to carry out essential routine activities. Neurons in the brain demand a constant supply of glucose, which depends on its extracellular concentration [54,112]. The brain is highly vulnerable to oxidative insults because of its high oxygen and glucose consumption rates, abundant lipid content, and relative paucity of antioxidant enzymes compared with other tissues [113]. 

**Figure 2:**
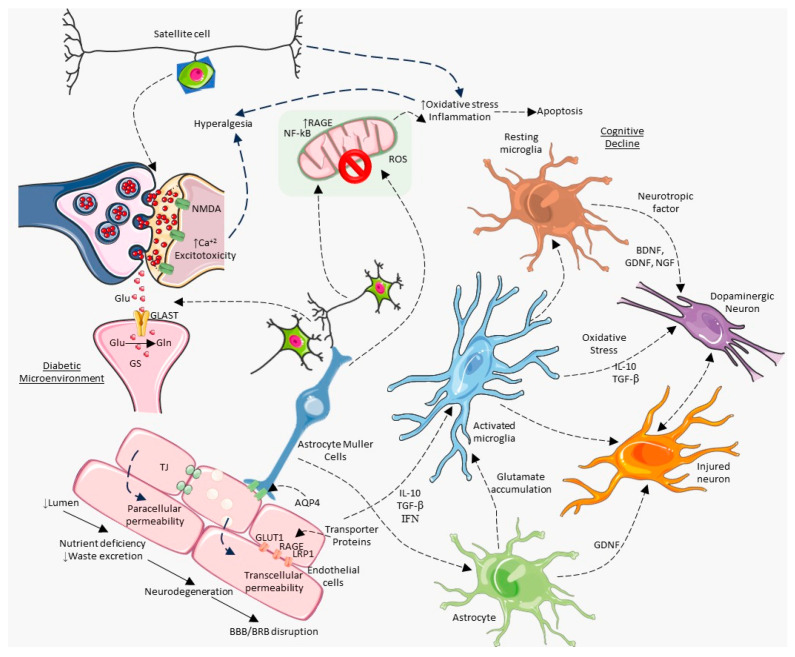
Diabetic microenvironment activates multiple neural cells, contributing to cognitive impairment and glial cell death.

High levels of blood glucose in the endothelial cells of the cerebral vascular system harm neurons and glial cells. The leading molecular pathologies that induce tissue damage due to hyperglycemia include the polyol pathway, which amplifies glucose influx, enhanced advanced glycation end products (AGEs), glycation of intracellular metabolic signaling proteins, robust activation of protein kinase C and its isoforms, and an overwhelming hexosamine pathway flux to increase N-acetylglucosamine-modified proteins [114]. Aldol reductase, the main enzyme in the polyol pathway [115], converts glucose to sorbitol and fructose using nicotinamide-adenine dinucleotide phosphate (NADPH), an essential cofactor that regenerates the powerful endogenous antioxidant glutathione. As hyperglycemia is exacerbated, accumulated glucose continues to be processed by excessive cofactors, resulting in elevated levels of reduced glutathione, oxidative stress, and polyol pathway flux. Hyperglycemia-induced increases in intracellular AGEs modulate cell signaling, gene transcription, and vascular pathology by reshaping extracellular and intracellular regulatory proteins. High blood glucose levels trigger the aberrant proliferation of diacylglycerol, a secondary messenger that activates the signaling cascade of protein kinase C and its isoforms. The anomalously activated protein kinase C enhances vascular pathologies by inducing proinflammatory conditions through the modification of gene expressions of inflammatory mediators such as endothelial nitric oxide synthase (a vasodilator), endothelin-1 (a vasoconstrictor), transforming growth factor-β, NF-κB, NADPH oxidases, and plasminogen activator inhibitor-1. As free glucose increases in hyperglycemia, the hexosamine biosynthesis pathway, a minor branch of glycolysis, becomes hyperactive. This results in the production of uridine diphosphate N-acetyl-glucosamine, a transcription factor modifier that alters gene expression, resulting in enhanced pathologies. Under hyperglycemic conditions, enhanced free fatty acid flux imposes oxidative stress on the mitochondria by increasing ROS, mainly superoxide ions, causing microvascular and macrovascular complications [116]. In the hyperglycemic brain, antioxidant defenders, such as superoxide dismutase, catalase, and glutathione peroxidase, are exhausted. Additionally, lipid peroxidation, glucose autoxidation, and low concentrations of reduced glutathione are major sources of oxidative stress in brain injuries [113,117]. Subsequently, cellular and mitochondrial oxidative stress can trigger apoptosis and/or necrosis [118], leading to the prevalence of neurogenesis inhibition [119]. The classical pathogenesis of cerebral dysfunction involves neuronal death and defective autophagy due to elevated glucose-induced mitochondrial oxidative stress, endoplasmic stress, and an inflammatory response against imbalanced metabolism. The dysfunction of glial cells (astrocytes, microglia, and oligodendrocytes) can lead to cognitive decline [120]. Figure 2 depicts the effects of a diabetic microenvironment and the involvement of several types of neuronal cells that may contribute to cognitive impairment in neurological illnesses. The diabetic condition influences the activation of detrimental pathways in neuronal cells. 

Indeed, both oxidative stress and inflammation are sensed by the master regulator NF-κB, which deliberately positions itself at the crossroads of both pathways, mediating events depending on the stimulus. The pathological role of NF-κB is also demonstrated in animal studies, as it is a critical molecule whose abnormal activation can cause neuronal apoptosis and impaired cognitive function [113]. Hence, dysregulated mitochondrial electron transport chain, ROS formation, mitochondrial energy metabolism dysfunction, and oxidative stress are the chief players in the hyperglycemic complications of the brain [121]. Recently, insulin resistance has been eccentrically exploited in the nervous system. Neurons, while insulin-independent, demonstrate insulin responsiveness, in contrast to insulin-dependent muscle and adipose tissues [122]. Insulin mediates mitochondrial metabolism through PI3K/Akt signaling [123]. Therefore, insulin resistance reduces Akt signaling and magnifies oxidative stress, leading to apoptosis, neurodegeneration, and consequent diabetic neuropathy [124]. Chronic cerebral complications involving ameliorated mental flexibility, cognitive impairment, and psychomotor function retardation may be accompanied by a metabolic malfunction. This malfunction results from impaired glucose homeostasis and insulin signaling caused by either insulin deficiency or insulin receptor desensitization. In conclusion, hyperglycemia depletes antioxidants and concomitantly leads to the accumulation of free radicals. These radicals activate redox-sensitive genes, alter the redox potential of cells, and cause tissue damage [113]. Thus, the underlying mechanisms of hyperglycemia-associated glucose neurotoxicity mainly include oxidative stress and protein glycation induced by elevated glucose levels in the brain. Notably, in addition to neurons, complementary glial cells indirectly affect glucose neurotoxicity [54,124,125]. 

High oxidative stress and redox imbalance, resulting from abnormal glucose metabolism under hyperglycemic conditions, promote protein aggregation and modifications, such as glycation. This process leads to the formation of intracellular advanced AGEs, particularly glyceraldehyde-derived AGEs. These AGEs subsequently affect signaling and energy production, potentially leading to cerebrovascular complications and AD [126,127]. Neurite degeneration arises from synaptic and neurotransmission inefficiencies, flawed energy metabolism, impaired membrane repolarization, faulty neurotransmitter synthesis or recycling, tau protein aggregation, and neurofibrillary tangles [128]. These catastrophic events can result in cognitive deterioration, astrogliosis, and neuroinflammation. Misfunctioning glial cells initiate antagonistic pathological neuron–glia interactions that create a “hostile” environment, impairing the functionality of neuronal cells, which can be correlated with the severity of the neurological deficit [5]. 

## 8. Diabetes, Cognitive Impairment, and Neurodegeneration

Diabetes is a complex chronic metabolic disorder characterized by high blood sugar levels, commonly referred to as hyperglycemia, which are a well-known risk factor for various complications, including neuropathy, nephropathy, angiopathy, and retinopathy [54,120]. Prolonged diabetes results in structural, neurophysiological, and neuropsychological alterations, along with multiple pathogenic factors associated with the pathogenesis of cerebral dysfunction [113,129]. Insulin deficiency in patients with type 1 diabetes negatively affects cognitive ability, intellectual functioning, memory, mental flexibility, attention, and psychomotor speed. By contrast, insulin resistance in T2D [130,131,132] results in impaired learning and immediate memory. In animal studies, corresponding results indicated inadequate insulin signaling through hippocampal receptors, influencing the regulatory processes of diet intake and cognition, mainly the long-term consolidation of information and memory function [129]. Physiological abnormalities in the brain alter neurotransmission, promoting neuronal loss, demyelination, cognitive dysfunction, vascular dementia, gliosis, AD, and depression in both experimental models and patients [133,134]. These electrophysiological and structural changes and impaired cognitive functioning are disruptive effects of hyperglycemia on the CNS [135], which account for cerebral complications referred to as “diabetic encephalopathy.” Diabetic encephalopathy is a term that specifies the impact of diabetes on the CNS, causing brain dysfunction and finally ending in mild to severe cognitive impairment. The term “diabetic encephalopathy” has been recently updated to “diabetes-associated cognitive decline”, as it perfectly describes a state of mild to moderate cognitive impairment, particularly psychomotor slowing and diminished mental flexibility not attributable to other causes [136]. Moreover, it is evident that diabetes-induced cognitive dysfunction negatively affects hippocampal integrity and long-term potentiation of synaptic plasticity in learning and memory processes [137]. 

The interaction of T2D and dementia has been extensively investigated in animal models. Animal models of diabetes, representative of insulin deficiency and chronic hyperglycemia, show unaffected glucose transport at the BBB, leading to high glucose concentrations in the brain. The anabolic hormone insulin regulates glucose homeostasis, energy metabolism, redox balance, osmolarity, and synaptic activity. Insulin and its IGF-1 receptors also control Aβ clearance and tau phosphorylation [138]. Therefore, insulin resistance increases the levels of amyloid precursor protein, senile plaques, Aβ accumulation, and tau hyperphosphorylation, initiating the formation of neurofibrillary tangles, the neuropathological hallmarks of AD [139].

The relationship between T2D and dementia is a crucial focus of current research. Studies have demonstrated that the onset of T2D may contribute to an increased risk of dysfunction in brain tissues, resulting in AD and other neurodegenerative diseases. Ramos-Rodriguez et al. highlighted central proliferation and neurogenesis disturbances in T2D in an animal model. Their findings in db/db mice (a model of diabetes) revealed age-dependent cortical and hippocampal atrophy and altered neurogenesis. These data suggest that T2D may underlie a few pathological characteristics that could exacerbate the features of dementia [140]. A similar finding from the same group suggested that T2D may promote numerous pathological and behavioral changes observed in dementia. These pathological changes have been observed in well-established models of diabetes, such as ob/ob and streptozotocin (STZ)-induced diabetic mice [141]. Type 1 diabetes rat models with chronic hyperglycemia and hypoinsulinemia have impaired hippocampal neuromodulation systems that regulate metabolism, synaptic activity, and memory performance [142]. Therefore, alterations in hippocampal physiological activities via BBB disruption, induced by changes in the concentrations of substrates and intermediate metabolites due to diabetes and metabolic disorders, articulate a close link between brain function and its bioenergetics [143]. Indeed, glutamatergic neurotransmission is robustly reduced in the brains of rats with streptozotocin-induced type 1 diabetes. Exclusively, cerebral cortical slices from streptozotocin-induced diabetic rats showed defective astrocytic glutamate clearance as a result of increasing glutamate levels in the synaptic cleft to induce excitotoxicity, a pathological process associated with neurodegeneration and motor dysfunction [144]. Furthermore, rats subjected to streptozotocin showed depleted nerve blood flow due to impaired conductance induced by prolonged diabetes [145]. Similarly, streptozotocin- or alloxan-induced diabetic rats modulated the secretion of certain hormones, such as dopamine, norepinephrine, and serotonin, which are fundamental regulators of normal brain function [146].

Diabetes may predispose individuals to AD, as demonstrated in the APP/PS1 mice model of AD. Diabetes, induced by STZ in an AD model, can aggravate AD by impairing working memory. In postmortem analyses of APP/PS1 diabetic mice, brain shrinkage, tau and amyloid-beta pathology, spontaneous bleeding, and elevated central inflammation were observed. Moreover, the study reported a change in Aβ soluble/insoluble levels towards more lethal soluble species in APP/PS1-STZ diabetic mice. APP/PS1-STZ animals also showed elevated phospho-tau levels and worsened inflammatory responses [147]. In an animal study, an interaction between diabetes and AD was observed in AD transgenic and ob/ob diabetic mice. The authors crossed Alzheimer’s transgenic mice (APP23) with two types of diabetic mice and analyzed their brain pathology. In double-mutant (APP+ -ob/ob) mice, the development of diabetes aggravated AD-like cognitive impairment without causing an increase in brain amyloid-β load. APP+ -ob/ob mice exhibited significant amyloid angiopathy and cerebrovascular inflammation, which is noteworthy. By contrast, compared with ob/ob mice, crossbred mice displayed an accelerated diabetes phenotype [148]. 

In recent years, numerous findings on maternal diabetes based on the studies in both humans and animals [149,150,151,152,153], indicates that maternal diabetes may be linked to the development of neurological diseases or disorders in offspring due to the activation of chronic inflammatory responses in the brain [154]. Perea et al. reported in a prospective study that prenatal exposure to gestational diabetes mellitus elevates the risk of attention deficit/hyperactivity disorder (ADHD) in offspring, while postnatal exposure was not identified as a risk factor for developing ADHD [155]. Camprubi Robles et al. reported a systematic review and meta-analysis comprising 12–15 studies based on the neurodevelopment of infants and children up to 14 years of age, combining data for children born to mothers with diabetes and without diabetes. There were 6140 children of different age groups from one to fourteen years of age. They found a significant decrease in scores of mental and psychomotor development in infants born to diabetic mothers at 1–2 years of age. Similarly, they reported a lower IQ in school-age children born to diabetic mothers, but the results were ambiguous due to high heterogeneity [156]. 

## 9. Conclusions

The most complex tissue in higher eukaryotes is the brain, which performs indispensable processes involving personality, cognition, and motor functions. Trauma, aging, and common metabolic disorders result in inadequate brain function, which poses a heavy burden on social and economic welfare. Unsuppressed hyperglycemia and metabolic derangements cause severe microvascular complications and multi-organ disorders. Both hyperglycemia and compromised cerebral insulin signaling lead to neurodegeneration, causing cognitive dysfunction and dementia. Currently, studies examining the effects of high blood glucose on the human brain and its function are attracting considerable attention. Metabolic changes, rather than being confined to metabolic organs, also negatively influence cerebral structure and function. Neurons are known to be catastrophically affected. However, glial cells are not merely used for anatomical support; rather, various types of glial cells are recognized for their involvement in a diverse array of essential roles in physiological homeostasis in the brain. Glial cell dysfunction can cause devastating impairment and pathologies that promote neurological and psychiatric illnesses. Thus, glial cells are clearly critical for brain function.
